# Influence of Inherent Surface and Internal Defects on Mechanical Properties of Additively Manufactured Ti6Al4V Alloy: Comparison between Selective Laser Melting and Electron Beam Melting

**DOI:** 10.3390/ma11040537

**Published:** 2018-03-31

**Authors:** Michaela Fousová, Dalibor Vojtěch, Karel Doubrava, Matěj Daniel, Chiu-Feng Lin

**Affiliations:** 1 Department of Metals and Corrosion Engineering, University of Chemistry and Technology Prague, Technická 5, 166 28 Prague, Czech Republic; vojtechd@vscht.cz; 2 Department of Mechanics, Biomechanics and Mechatronics, Czech Technical University in Prague, Zikova 1903, 166 36 Prague, Czech Republic; karel.doubrava@fs.cvut.cz (K.D.); matej.daniel@fs.cvut.cz (M.D.); 3 Metal Industries Research & Development Centre, Kaonan Highway 1101, 811 60 Kaohsiung, Taiwan; chiufeng@mail.mirdc.org.tw

**Keywords:** Ti6Al4V, selective laser melting, electron beam melting, mechanical properties, fatigue

## Abstract

Additive manufacture (AM) appears to be the most suitable technology to produce sophisticated, high quality, lightweight parts from Ti6Al4V alloy. However, the fatigue life of AM parts is of concern. In our study, we focused on a comparison of two techniques of additive manufacture—selective laser melting (SLM) and electron beam melting (EBM)—in terms of the mechanical properties during both static and dynamic loading. All of the samples were untreated to focus on the influence of surface condition inherent to SLM and EBM. The EBM samples were studied in the as-built state, while SLM was followed by heat treatment. The resulting similarity of microstructures led to comparable mechanical properties in tension, but, due to differences in surface roughness and specific internal defects, the fatigue strength of the EBM samples reached only half the value of the SLM samples. Higher surface roughness that is inherent to EBM contributed to multiple initiations of fatigue cracks, while only one crack initiated on the SLM surface. Also, facets that were formed by an intergranular cleavage fracture were observed in the EBM samples.

## 1. Introduction

Over the past 20 years, titanium and titanium alloys production has developed more rapidly than perhaps any structural material in the history of metallurgy. That enabled the use of titanium materials in many safety-critical structures, such as those in aircraft, engines, or biomedical applications. Nevertheless, because of the high price of titanium and its costly and difficult manufacturing, there is still a focus on cost-effectiveness and reduction of product development time. Therefore, there is a high demand for light-weight materials of high specific strength and high quality, and a quest for pertinent technological approaches [1,2].

Titanium itself has a high strength-to-weight ratio, and, with proper design, it can meet the above-mentioned requirements. Nowadays, additive manufacturing (AM) seems to be the most promising technological approach to prepare sophisticated light-weight structures easily and cost-effectively [3]. When compared to conventional casting, forging, and machining, AM enables the direct and rapid production without a need for special molds, tools, or dies. Parts can be manufactured right in the final form based on a computer-aided-design (CAD) model and can be designed in very complex external shapes, but also with complex internal structures [4]. 

Lately, there have been very intensive studies in the field of metallic additive manufacturing (MAM) research [5]. Three categories of MAM working with a different material supply have been already developed. Among powder-bed technologies that build product by the selective melting of a metallic powder raked across a building plate in many successive thin layers, Selective Laser Melting (SLM) and Electron Beam Melting (EBM) are the leading techniques. Also, other terms appear in the literature, such as Direct Metal Laser Sintering (DMLS), Laser Metal Fusion (LMF), or LaserCUSING, etc., but these are basically synonyms of SLM given by different machine manufacturers. In powder-feed systems, the starting material is also a metallic powder, but it is conveyed through a nozzle and is melted before its deposition onto a building plate. This category is represented by Laser Metal Deposition (LMD)/Laser Engineered Net Shaping (LENS)/Laser cladding [6]. Wire-feed systems are based on the deposition and welding of a metallic wire into a final three-dimensional (3D) structure. These systems boast with the highest deposition rates, but are only suitable for less demanding applications. For demanding applications, powder-bed systems are the most suitable, because they provide a high resolution and maintain a dimensional control [7].

For all of the individual MAM techniques, microstructure evolution, mechanical properties in static and dynamic loading, the influence of processing parameters on relative density, the influence of building orientation, and many other issues have been investigated for different metallic materials in hundreds of research papers published during few last years. However, despite a very rapid development of MAM and the intensive effort of its extension into a serial production, reliable mechanical properties and clearly defined quality are still necessary to be investigated and achieved before the industrial use [7,8,9,10,11]. 

As SLM and EBM are the most promising techniques for high-quality titanium manufacture, these are the most discussed. Both techniques work on the same principle with the main difference being the use of a laser or an electron beam to selectively melt a powder material. Nevertheless, the use of different energy sources is accompanied by different processing conditions, and subsequently by characteristic features [12]. In EBM, a vacuum is needed to avoid the deflection of an electron beam by gas molecules, whereas SLM process is conducted in an inert gas atmosphere. While powder particles directly absorb the heat energy from photons of a laser beam, in EBM, electrons penetrate into powder converting their kinetic energy into thermal energy to melt the powder. An electron beam has a more diffuse spot size than a focused laser spot. The larger spot size, along with the larger size of powder particles, impede powder particles repelling each other due to charging by the electron beam. As a result, the minimum feature size, resolution, and surface finish of EBM are typically larger than for the SLM process. On the other hand, the EBM process is far more energy-efficient than the laser technology, and the vacuum supports the processing of reactive metals [13]. Therefore, for a particular type of application, it is important to compare SLM with EBM in terms of part quality, mechanical performance, surface properties, resistance to corrosion, and high temperatures. In our paper, we focused on the comparison of tensile behavior and fatigue performance of the Ti6Al4V alloy that was prepared by SLM and EBM AM and correlated mechanical properties with inherent microstructures and surfaces.

Although there have been several papers published in last few years dealing with the comparison of the Ti6Al4V alloy prepared by SLM and EBM technology, the discussed results are not always consistent. That is mainly given by a broad parameter variety affecting the material performance. Rafi et al. [14] were one of the first to publish a study comparing SLM and EBM technologies in the processing of the Ti6Al4V alloy. Primary as-built products were tested. The higher tensile strength, lower ductility, and higher fatigue strength of SLM products were attributed to a martensitic microstructure. Chan et al. [15] focused especially on the comparison of the two AM techniques with conventional casting and rolling. Zhao et al. [16] compared SLM and EBM on as-built samples too and studied the influence of a part size, part orientation, and HIP treatment. It was concluded that: the part size has influence only on EBM, affecting α-lamellae thickness; vertical orientation results in higher mechanical properties than horizontal; and, HIP positively influences the fatigue strength by defect closure. Gong et al. [17] carried out a comparison of both the technologies, also in an untreated state. Formation of defects and their effects were the main object of interest. Gretemeier et al. [18] compared the fatigue of AM Ti6Al4V, but in annealed or HIPed state. Günther et al. [12] focused on the comparison of fatigue performance. SLM was followed by heat treatment, so that a similarity in microstructure with EBM was achieved and the microstructure influence could be neglected. Because all specimens were electropolished, defects were the only dominating factor remaining, on which Günther et al. focused in detail. However, the influence of an inherent surface was not studied. That is particularly important for the case of porous structures that cannot be machined.

Our goal was to provide a complex study of Ti6Al4V that was obtained by both technologies, SLM and EBM, in a similar state of the microstructure. That is an important fact, because each technology works differently, although the general principle is the same. As high scan speeds that are related to the electron beam technology enable preheating of every layer prior to melting, EBM processing is performed at elevated temperatures >973 K. As a result, temperature gradients and local cooling rates are reduced. While fast cooling in SLM results in the formation of α’-martensite and high internal stresses, slower cooling in a vacuum leads to α/β transformation [19]. Therefore, in our study, SLM was combined with a post-process heat treatment, while EBM was studied in the as-built condition. Microstructural features and porosity, both are factors influencing mechanical performance, were very close, so that the characteristic features of both technologies, such as surface condition or defect type, were the most pronounced and could be compared.

This paper brings a detailed microstructural analysis to show that the selected processing route yielded similar results. The material performance is characterized via tensile and fatigue tests. A fractographic analysis is used to demonstrate the influence of inherent surface roughness and internal defects.

## 2. Materials and Methods

### 2.1. Materials

**Figure 1:**
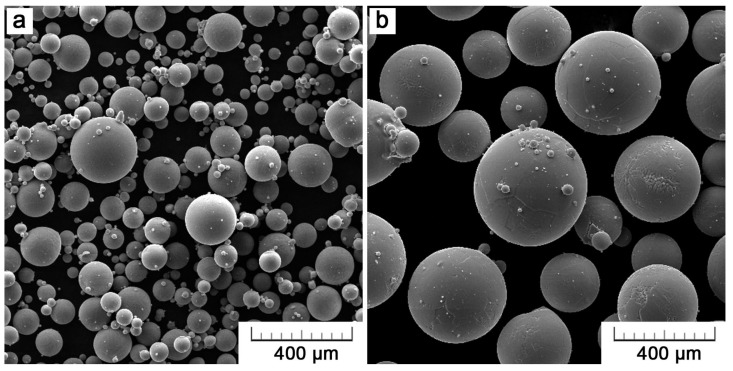
Morphology of Ti6Al4V powder for: (**a**) Selective Laser Melting (SLM) and (**b**) Electron Beam Melting (EBM) technology.

Testing parts of the Ti6Al4V alloy were produced using an M2 Cusing SLM machine (ConceptLaser, Lichtenfels, Germany) and an Arcam Q10 EBM machine (Arcam, Mölndal, Sweden). Powders of a gas-atomized Ti6Al4V alloy were provided according to the recommendation of each machine manufacturer by Dentaurum and AP&C, for SLM and EBM, respectively. The average particle size of rematitan^®^ CL (Dentaurum, Ispringen, Germany) was 30 µm. EBM uses larger particle size, with average at 75 µm (45–105 µm, AP&C). The morphology of both powders, as observed by a scanning electron microscope (TESCAN VEGA-3 LMU, Brno, Czech Republic), is shown in Figure 1. Almost perfectly spherical powders were prepared by gas atomization. Small satellite particles that formed during the atomization process are clustered around larger particles.

**Figure 2:**
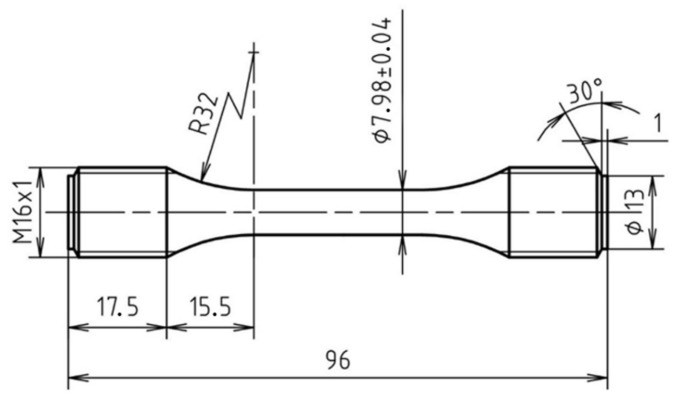
Technical drawing of specimens designed for fatigue tests.

Specimens conforming to the standards for tensile (CSN EN ISO 6892-1) and fatigue (CSN EN ISO 12110-1) testing were fabricated. The geometry of the fatigue specimens is given in the technical drawing in Figure 2.

In the SLM process, the specimens were produced with a laser beam of 200 W power with a spot size of about 200 µm. Scanning speed was set to 1250 mm s^−1^, hatching space to 80 µm. The parts were built vertically on a Ti6Al4V building plate preheated to 473 K in 30 µm thick layers by using an island scanning strategy (with island size of 5 × 5 mm^2^). The building chamber was supplied with a protective argon atmosphere (technical argon, 4.6 purity). In the EBM process, an electron beam with a 100 µm spot size was generated at a voltage of 60 kV. Related to the larger powder size, the layer thickness was set to 50 µm. For each layer, a pre-scan at a higher beam current (30 mA) and a higher scanning speed (10,000 mm s^−1^) was performed to keep all of the material preheated up to 1013 K. Selective melting was done by a continuous scanning of the electron beam (15 mA current) at a speed of 4530 mm s^−1^ in adjacent lines, with a hatching space of 0.2 mm. The melting process was carried in an evacuated chamber.

The testing parts were kept in the as-produced state, without any surface treatment. For the SLM-fabricated parts, additional heat treatment was applied to minimize the internal stresses and obtain a comparable microstructure as the EBM-produced parts. The finished parts along with the building plate were placed into a vacuum furnace, heated up to 1093 K in 4 h, kept at this temperature for 90 min, and cooled down slowly. 

### 2.2. Surface and Microstructure Characterization

The surface morphology of the as-produced parts was observed in a scanning electron microscope TESCAN VEGA-3 LMU (Brno, Czech Republic) equipped with Oxford instruments INCA 350 EDX analyzer (Abingdon, UK). Surface roughness, represented by mean roughness depth Rz, was assessed by Mitutoyo SurfTest SJ400 (Kawasaki, Japan) (JIS B 0601-2001 standard, 2.5 mm cut-off length, 10 measurements). For a microstructural analysis, longitudinal and transverse sections were prepared by grinding on SiC papers, mechanical polishing on diamond pastes, and final chemical-mechanical polishing in silica suspension supplemented with 20 vol% of hydrogen peroxide. The polished sections were first subjected to optical microscopy (Olympus PME3, Tokyo, Japan), along with an image analysis (ImageJ software, version 1.51, NIH, Bethesda, MD, USA) to determine the porosity representing fabrication defects. More than 25 images capturing the whole longitudinal section were analyzed. After etching in Kroll’s reagent, microstructures were observed. Microstructural dimensions were also evaluated by image analysis.

### 2.3. Evaluation of Mechanical Properties

Static mechanical properties of the SLM and EBM processed parts were evaluated in uniaxial tension. Tensile tests were performed according to the CSN EN ISO 6892-1 standard using a LabTest 5.250SP1-VM (LaborTech, Opava, Czech Republic) universal loading machine at room temperature with a strain rate of 0.001 s^−1^. For statistical purposes, three specimens were measured. 

Fatigue tests were carried out using a resonant electromagnetic testing machine Amsler HFP10 (Zwick Roell, Ulm, Germany) that was equipped with computer control enabling crack detection based on the frequency drop. A sine-wave fully reversed load (R = −1) was applied with a frequency of 100 Hz. To obtain S-N fatigue curves, 15 specimens were loaded at selected stress amplitudes (σ_a_ = 100–600 MPa) and the number of cycles to failure was determined. For the specimens that remained undamaged, testing was terminated after 10^7^ cycles. The fatigue tests were carried out at room temperature in the air.

Broken specimens after both tensile and fatigue test were subjected to a fractographical analysis by SEM. 

## 3. Results

### 3.1. Additively-Manufactured Ti6Al4V

#### 3.1.1. Microstructure

**Figure 3:**
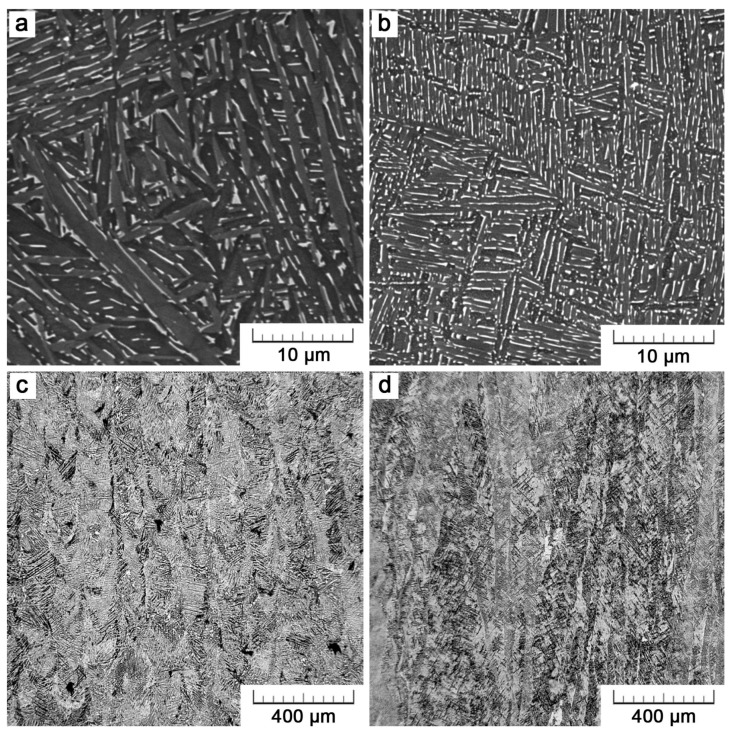
Micrographs showing a fine two-phase lamellar morphology and elongated grains (parallel to the building direction) of Ti6Al4V manufactured by SLM (**a**,**c**) and EBM (**b**,**d**).

Figure 3 displays microstructures of Ti6Al4V alloy that was manufactured by the additive technology. Figure 3a,c belong to a part manufactured by SLM, the remaining two to an EBM part. For both parts, the microstructure exerts similar lamellar morphology consisting of thin α-lamellae (dark) that are separated by a small amount of interlamellar β phase (bright). In lower row of micrographs obtained at a lower magnification, long columnar grains can be distinguished. These are prior-β grains growing across several layers in an epitaxial manner. In EBM, prior-β phase within these grains transforms into a lamellar mixture of α+β during cooling. Owing to the high cooling rates in SLM (up to 10^6^ K/s [14]), martensitic needles are formed in the as-built specimens. Nevertheless, after the application of post-process heat treatment at 1093 K below the β transus, these martensitic needles decompose into α+β lamellae. In Figure 3c, also some porosity can be noticed. Nevertheless, a very good relative density higher than 99.5% was achieved. Results of the porosity assessment are summarized in Table 1. 

The differences between Ti6Al4V microstructures that are prepared either by SLM or EBM are of a dimensional nature. At first sight, the EBM microstructure is finer. Not only the α-lamellae thickness is two times lower, but also the thickness of prior-β grains is lower. In the SLM parts, the prior-β thickness distribution is more regular. In EBM, more than a half of the grains are very thin, in the range of 20–50 µm, but there are also a few over 100 µm. The exact values obtained by image analysis are given in Table 1.

#### 3.1.2. Surface

**Figure 4:**
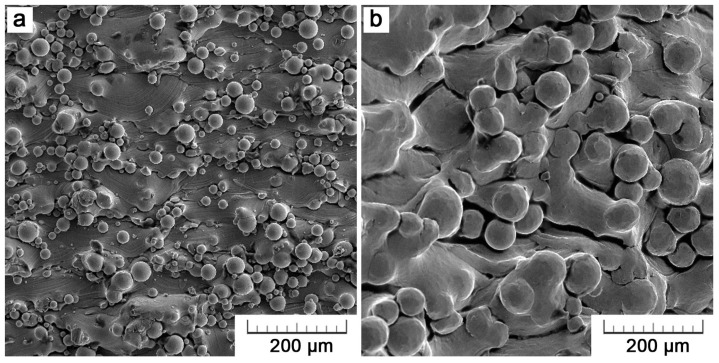
Surface morphology of Ti6Al4V specimens manufactured by (**a**) SLM and (**b**) EBM.

The surface of the as-built parts is shown in Figure 4. Both of the 3D printing technologies result in surfaces that are covered with a certain amount of adhering unmelted particles initial powders. While some particles are loosely bound, others are partially melted. The problem of adhering particles is a generally known drawback of powder-bed techniques and a perfectly smooth surface can be never achieved. Even so, the surface quality can be influenced by process parameters set-up. The adhesion of unmelted or partially melted particles is brought by the process principle itself and can be explained by the combination of thermal diffusion and partial melting of boundary particles by a contour laser/electron beam track setting bounds of the part in each layer. The thermal diffusion occurs between a loose powder in a powder bed and a solidified material underneath due to a big temperature difference. It leads to powder particles sticking to a product surface [20].

EBM surface exerts a higher roughness (Rz of 71 ± 22 µm as compared to 31 ± 12 µm), which is given by the use of a coarser powder (~75 µm vs. ~30 µm). Also, the higher power of the EBM process itself assists higher unevenness. That is an EBM drawback that should be taken into account before processing. Parts are usually designed larger and are then machined into the desired size to overcome dimensional inaccuracies [21,22]. However, such an option cannot be taken into account when porous structures are about to be designed [23].

In Figure 4a, overlapping arcs can be distinguished. The arc-bounded domains represent solidified melt pools that are formed by a single laser track. The arc radius (about 80 µm) corresponds to the depth of melt pools but is probably enlarged by the effect of gravity. In EBM, there are locally large depressions in between gradually solidified areas (Figure 4b) because solidification is the fastest at part surface and so a melt fail to fuse with a previous layer properly. These depressions disrupt the continuity of a surface and are unfavorable in terms of fatigue.

### 3.2. Mechanical Properties

#### 3.2.1. Static Properties

**Figure 5:**
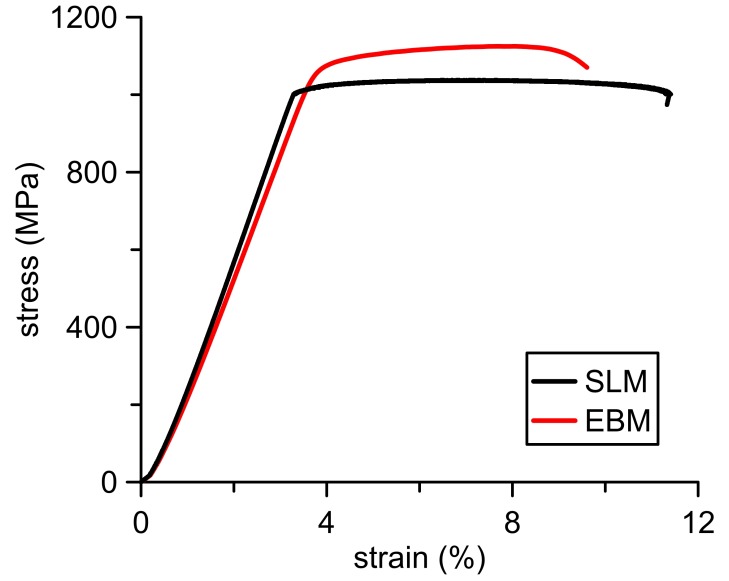
Exemplary stress-strain curves for EBM and SLM tensile specimens.

Exemplary stress-strain curves in Figure 5 show the behavior of the 3D-printed Ti6Al4V alloy. High slopes of linear parts testify a high modulus of elasticity. Following plateaux, when almost constant stress leads to an irreversible deformation, indicate plasticity of the material. Regarding comparable two-phase microstructures of the SLM and EBM parts, tensile properties show very similar values (Table 2). Owing to the finer microstructure, the EBM specimens exerted only a slightly higher ultimate tensile strength (UTS) of 1132 ± 11 MPa and a yield strength (YS) of 1074 ± 14 MPa. In relation to that, elongation was 10% lower.

#### 3.2.2. Fatigue

**Figure 6:**
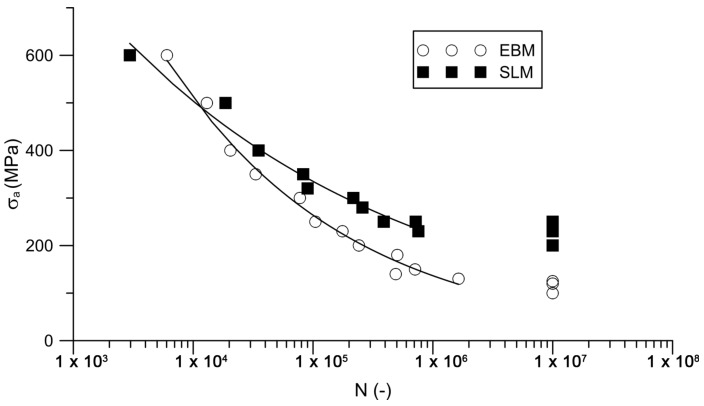
S-N curves of SLM and EBM Ti6Al4V.

Figure 6 shows S-N curves for Ti6Al4V that were manufactured by both additive technologies, EBM and SLM. The curve for SLM is positioned above the EBM curve reflecting that the SLM material exerts a higher fatigue life. The fatigue strength of the SLM material is 220 ± 24 MPa, while 115 ± 13 MPa for EBM. 

### 3.3. Fractography

**Figure 7:**
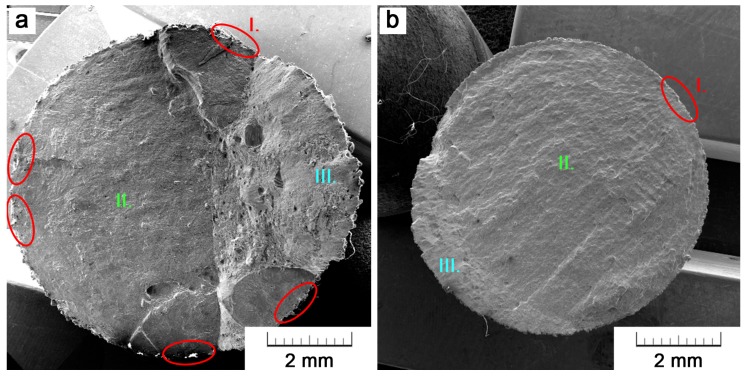
Comparison of fatigue fracture surfaces of (**a**) EBM and (**b**) SLM specimen. Areas I. (in red) represent initiation sites, II. represent areas of fracture propagation (in green) and III. is the final fracture area (in blue).

A fractographic analysis was carried out to reveal the reasons of different fatigue lives of the specimens that were manufactured by a different method of the additive technology. In this paper, two specimens are shown for an illustration (Figure 7). Particularly, it concerns specimens that are loaded at a stress amplitude of 230 MPa. The SLM specimen failed after 756,620 cycles, while EBM after 175,649 already. Nevertheless, the observed trend was the same for all of the specimens.

Figure 7 brings the comparison of both fracture surfaces on a macroscopic scale. Crack initiation sites are marked with red color (area I.), green color represents the areas of a slow crack propagation (area II.), and the areas of a rapid final fracture are marked in blue (area III.). The area of fracture propagation occupies the majority of the fracture surface. Nevertheless, there is an obvious difference between EBM and SLM where this area occupies only 3/5 of the whole fracture area as compared to 7/8. The rest is a final fracture area that occurred when the load-bearing area was already insufficient. This is the first sign that the EBM specimen cracked earlier. The main evidence is, however, the detection of five sites on the specimen surface, where a crack initialized. At the first stage, several cracks initialized and propagated to merge into one marginal crack. Therefore, the fracture was easier to occur and the fatigue life was shorter. What concerns the SLM specimen, there was only one initiation site. 

**Figure 8:**
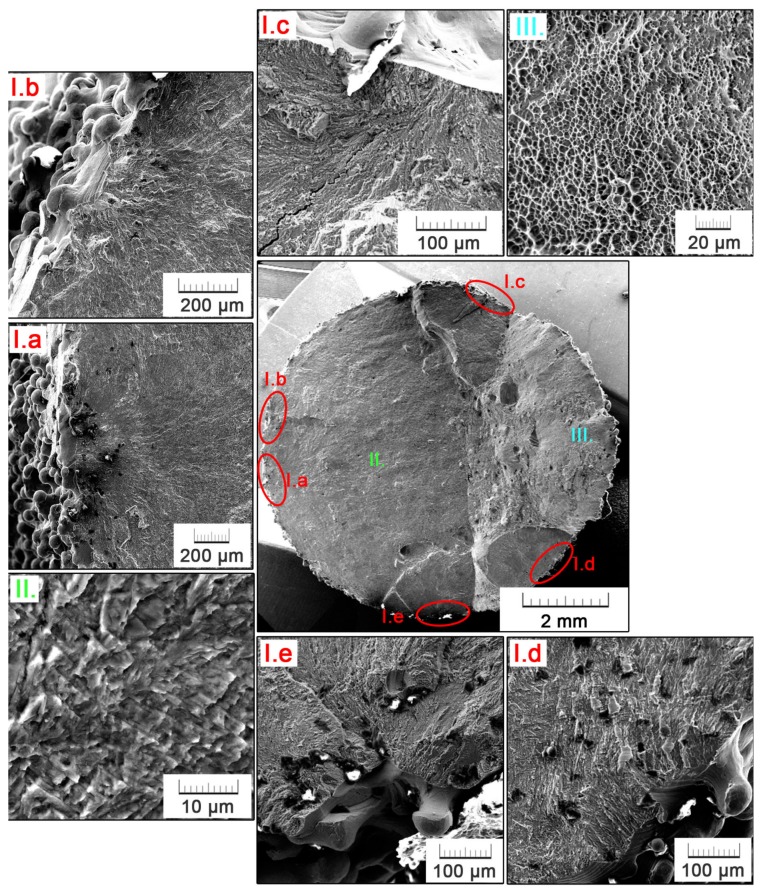
Details of crack initiation sites (**I.a**–**e**), crack propagation (**II**.) and final fracture (**III**.) areas on the fracture surface of the EBM fatigue specimen.

The fractographic analysis of the EBM specimen is depicted in detail in Figure 8. Five main initiation sites are represented by surface irregularities, manifesting in a notch effect (Figure 8I.a–e). Areas that are adjacent to the location of crack initiation are contaminated by dust and other particles, which penetrated into the crack entry during the tensile component of cyclic loading. In Figure 8I.a, a typical flabellate propagation of the fatigue crack can be observed. The area of crack propagation is further magnified in Figure 8II. A fragile fracture nature is evidenced. Also, characteristic ‘beachmarks’, formed as a result of cyclic loading when the crack opens and propagates only in periods of tensile stress action, can be distinguished. Figure 8III. shows the final fracture area, which, contrarily to the fatigue fracture, exerts a clearly ductile nature with a fine dimple morphology.

**Figure 9:**
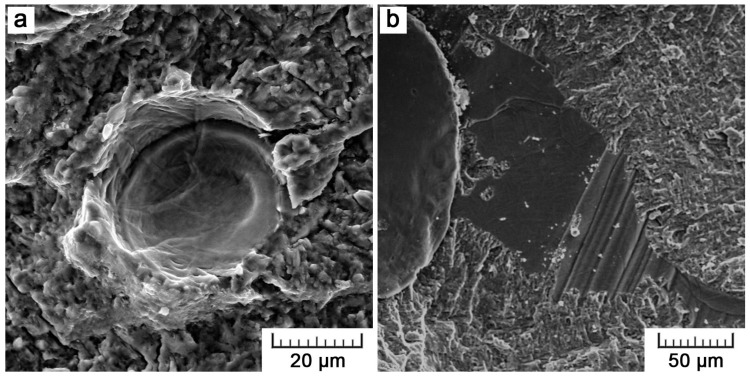
Defects observed in the fatigue crack propagation area of EBM specimen: (**a**) gas bubbles and (**b**) facets.

Figure 9 documents defects that were observed within the area of crack propagation (area II.). It concerns particularly spherical voids of 30–50 µm in size (Figure 9a). Despite their low size, these defects are relatively numerous, homogeneously distributed across the whole fracture surface, so that they may promote the crack propagation and negatively affect the fatigue life. The facets that are shown in Figure 9b are the second type of defects. In these areas, the material was ruptured intergranularly, so that a smooth surface is observed. Such facets reached up to several hundreds of micrometers. Therefore, their negative effect was even more pronounced. As many large facets were present in the final fracture area, it can be supposed that they probably contributed to the earlier rupture of the EBM specimen. To assess the chemical composition of these facets, the EDX analysis was accomplished. Within the facets, a significant enrichment in vanadium was recorded. While the constitutive material consists of 5.2 ± 0.8 wt % of Al and 3.8 ± 0.1 wt % of V, the contents of these alloying elements in the facets were determined to be 6.5 ± 0.4 wt % and 13.0 ± 0.8 wt %, respectively. Due to the analysis resolution, the quantitative data may not be exact, but may be slightly disturbed by surroundings. Nevertheless, regarding the high vanadium content, it can be supposed that the facets are facets of β-phase.

**Figure 10:**
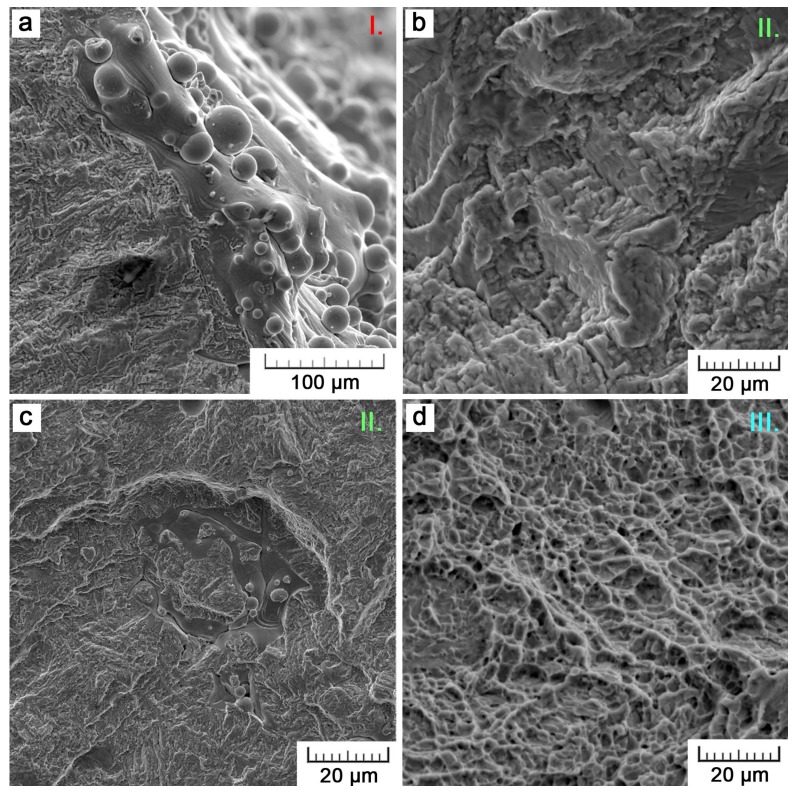
Details of the fracture surface of the SLM fatigue specimen: (**a**) initiation site (area I.); (**b**) crack propagation (area II.); (**c**) defect; (**d**) final fracture (area III.).

The fractography of the SLM specimen is depicted in Figure 10. As it was shown in Figure 7, there was only one initiation site on the surface of the SLM specimen, by contrast to the EBM specimen. This site is captured in Figure 10a. Similarly to the EBM fracture surface, the area of crack propagation exerts a fragile nature and the final fracture area a ductile nature. Nevertheless, the fracture morphology is generally coarser, which can be related to the coarser microstructure (see Table 1). Also, the SLM technology suffers from the formation of certain defects. These defects are, however, of a different origin (Figure 10c). They are caused by insufficient melting due to a not optimal set of process parameters. Using an island scanning strategy, single islands are scanned and melted by a laser beam in a random order. As a result of insufficient melting, islands do not fully interconnect with neighboring islands or with a previously solidified layer when they solidify. Although, in some locations of the fracture surface these defects were relatively large, there were less of them. These defects do not seem to initiate fatigue cracks but facilitate their propagation.

## 4. Discussion

### 4.1. Microstructure

Although the preparation of the compared parts was not the same, the resulting microstructures were similar. The reason is following:

In SLM, a powder is selectively melted by a laser beam and solidifies rapidly after the laser moves away. The cooling speed is about 10^4^ K/min [24]. During the process, a building plate is usually preheated up to 473 K, but the rest of the building chamber is not heated. As a result, there is a very high thermal gradient that is associated with the very high cooling rate. Due to that, no diffusion is possible and a displacive transformation takes place. The melt transforms into a nonequilibrium martensitic phase. Because of a high stress in the crystalline lattice, the resulting parts suffer from a low plasticity. Therefore, an appropriate heat treatment is usually applied after SLM. When heated up to 1093 K and cooled down slowly, the martensitic phase transforms into a mixture of α and β phases. The demanded result is a significant increase in ductility [25]. 

In EBM, a powder is also selectively melted, but by an electron beam of a high power. During the process, every layer is first preheated by a prescan of a lower power and after that the main melting scan follows. As a result, the fabrication is performed at powder-bed temperature above the martensitic transformation temperature (Ms = 848 K [26]), what favors the formation of α and β phases instead of α’ martensite [14].

The only difference observed was the fineness of α lamellae within the α + β columnar grains. The explication may be following: In EBM, the material was kept at an elevated temperature of 1013 K. As the melting of each layer is accompanied by the re-melting of several preceding layers and thermal affection of the surrounding material, the temperature of the already solidified material easily surpasses 1243 ± 50 K [27], which is the β-transus temperature. Therefore, a repetitive transformation β → α + β occurs. Resultant intense α-phase nucleation leads to a very fine lamellar microstructure. The principle of cyclic annealing around the transformation temperature can be used beneficially for microstructure refinement. e.g., in ref. [28], the advantage of solid state phase transformations was used for the refinement of the lamellar microstructure of γ-TiAl-based alloys. Similarly to EBM, previously-solidified material is also affected in SLM. Nevertheless, due to the absence of preheating, room temperature in a building chamber leads to very high cooling rates. Therefore, very fine martensitic needles are formed after SLM build-up. Subsequently, a thermal treatment that follows causes phase transformation and the coarsening of the original needles. During heating α phase nucleates along the martensitic needles, and, as martensitic phase is oversaturated with vanadium, V atoms are expulsed from newly created α lamellae and β phase is formed in the interlamellar space [29]. 

### 4.2. Mechanical Properties

#### 4.2.1. Static Properties

Both types of additively-manufactured specimens exerted very high strength properties in tensile loading. Both overpassed the properties of conventionally fabricated Ti6Al4V alloy (as it is shown in our previous study for SLM [30]). That is attributed to the high cooling rates leading to very fine microstructures. Conversely to the strength, the additively manufactured titanium alloy suffers from low plasticity (elongation). The internal defects acting as stress concentrators and high residual thermal stresses resulting from large thermal gradients are the cause [29].

When comparing the mechanical performance of SLM and EBM samples, there are several influences that are involved. First, related to a finer microstructure, EBM can be expected to reach higher values of strength and lower elongation. However, the material strength and also plasticity are negatively impacted by the additively manufactured surface. Therefore, in EBM, the strength contribution of the finer microstructure is suppressed by a higher surface roughness that promotes stress concentration. Also, the porosity plays its part. The theoretically higher plasticity of SLM samples showing a larger grain size is reduced by their higher porosity. Consequently, both of the technologies provide samples of comparable properties.

Also, other authors obtained similar properties as we did, but the exact values are dependent on many factors, such as processing parameters influencing a part density, orientation, size, or a particular regime of heat treatment. e.g., Zhao et al. [16] showed that YS and UTS change slightly with the sample diameter as α’-needles/α-lamellae size changes. Vrancken et al. [29] published variances in mechanical properties of SLM Ti6Al4V with different heat treatment regimes. Table 3 brings a summary of tensile properties that were obtained by other authors. Only papers where sample preparation, resulting microstructure and surface, were comparable to our work are listed. Generally, with respect to a wide range of possible process parameters settings, the values of mechanical properties can vary widely for both SLM and EBM. There is not any significant difference; for both technologies, mechanical properties overlap. YS ranges from ~860 to 1100 MPa, UTS reaches values from ~960 to 1130 MPa. In comparison to the data of other authors, our EBM samples reached the highest strength. SLM samples reached rather average values due to their higher defectiveness. In terms of material plasticity, EBM samples tend to reach higher elongation than SLM due to the higher process energy and lack of inappropriate fusion defects, but again the scattering of the values is very large.

Depending on particular processing conditions, AM can yield in slightly lower, comparable, or even higher static properties than a conventionally produced wrought alloy (Table 3).

#### 4.2.2. Fatigue

Despite a similar behavior in static tensile loading, the fatigue test showed a significant difference between both technologies. The specimens that were manufactured by EBM exerted a lower number of cycles until fracture than those that were manufactured by SLM. Only for the highest amplitude, the fatigue life of both EBM and SLM specimens was comparable. The difference became more and more pronounced with lower stress amplitudes in high-cycle fatigue. For the specific case of fatigue testing at the stress amplitude of 230 MPa, the difference in fatigue life was even fourfold. 

Although the EBM specimens seem to be more suitable for cyclic loading in terms of microstructure, our study showed that the effect of microstructural features and defects is negligible when compared to the effects of a surface. We reported finer prior-β grains, finer α-lamellae, and lower average porosity (Table 1), all factors contributing to a better resistance to fatigue. Even so, the results of fatigue tests were opposite. 

The fractographic analysis of both compared fracture surfaces revealed that several inhomogeneities on the EBM specimens surface acted as initiation sites of fatigue cracks. Generally, the cause of the observed phenomenon is thus the rougher surface that is yielded by EBM. While SLM uses the powder of an average particle size of 30 µm as the input material, EBM works with a coarser fraction of 80 µm in average. If the as-built surface is not further treated, as it was in this study, a relatively high amount of powder particles adhere to it. There have been many studies attesting the surface influence [15,39,40,41]. For SLM, it was shown that as-built specimens exert about 60% lower endurance limit than the polished ones. For polished specimens, the fatigue limit of about 500 MPa is comparable to the standard value for wrought Ti6Al4V [39]. In EBM, the influence of the inherent surface is even more pronounced as it can reduce the fatigue limit by up to 75% [42].

Secondary causes are internal defects that are formed during part build-up. In polished specimens, where the detrimental influence of as-built surface is eliminated, internal defects have been found to be the sites of crack initiation [43]. 

In EBM, two defect types were observed–spherical pores and flat facets. Spherical voids are the result of gas bubbles formed by gas entrapment in the Ti6Al4V melt and impossibility of its escape during rapid solidification. Although the EBM process is carried out in a vacuum, it probably concerns a gas that is enclosed in between powder particles in a powder-bed or a water vapor that is formed by the evaporation of moisture adhering to powders and walls of the working space [44]. Low amount of gas is even entrapped in the particles of starting powder formed by gas atomization process [45,46]. Flat facets result from a cleavage fracture. Zuo et al. [47] showed that in a ‘basketweave’ microstructure of Ti6Al4V, internal fatigue cracks initiate at α-β interfaces or prior-β grains boundaries because the shear processes parallel to the lamellar interfaces are relatively easy. As the hcp α phase is more brittle than the bcc β phase, slip starts first in β phase and dislocation arrays are piled up at α-β interfaces. As a consequence, long interfaces of α/β lamellae in a ‘basketweave’ microstructure become ‘weak’ sites in which internal cracks may initiate. Moreover, when lamellae are very thin, the whole colony of α-lamellae behave as one grain, so the grain boundaries may also be the sites of crack initiation. Especially boundaries that are oriented in the direction of maximum shear stress are the most critical.

In SLM, a different type of defects occurred. These are lack-of-fusion defects manifesting irregular shape, smooth surface, and sometimes containing enclosed unmelted powder particles. These defects are caused by the local disturbances during the melting process [40]. Due to an insufficient energy input that is given by an unideal set-up of processing parameters (such as a laser power, scanning speed, layer thickness, hatching distance, or selected scanning strategy), individual hatch lines or layers may not fuse sufficiently so that voids that are elongated in the scanning direction may be formed [48]. Thijs et al. [49] observed the formation of such pores with a certain order and attributed that to an accumulation in powder denudation around a melt pool within a layer and an accumulation of the surface roughness across layers. Nevertheless, fractography did not reveal crack initiation at these defects.

Although the overall porosity of EBM fatigue specimens was assessed to be lower (see Table 1), it does not necessarily need to reflect a lower impact on a fatigue life. In the EBM specimens, the overall porosity that was determined by image analysis corresponds only to spherical voids formed by gas entrapment. However, these voids did not significantly express themselves in fatigue. They only slightly reduced the load-bearing area and could facilitate crack propagation. But, there were defects of a greater harmful influence that were expressed only after the loading. Large interfaces between grains that were formed by β phase supported stress concentration and led to an intergranular cleavage fracture forming large facets on the fracture surface. In Günther’s et al. [12] work, it was even determined quantitatively by using Murakami’s model [50] that the stress concentration at such facets is higher than at lack-of-fusion defects in SLM specimens. Therefore, this type of defects also further supported the lower fatigue strength of the EBM specimens.

There are not many results to be found in the literature which would be comparable to our study. SLM has been studied predominantly in the as-built state with martensitic microstructure. If samples were stress-relieved, they were machined at the same time [15,16]. Similarly to us, Greitemeier et al. [18] obtained fatigue limit of 200 MPa for SLM and 150 MPa for EBM when the testing specimens with inherent surface roughness, but both annealed for 2 h at 983 K. A significant increase in fatigue limit was registered after surface effect elimination (475 and 270 MPa, respectively). Finally, additional hot isostatic pressing (HIP) treatment united fatigue performance of both technologies. Kahlin et al. [42] also tested fatigue with an as-built surface. Here, HIP did not influence the fatigue limit significantly (~180 MPa before and ~200 MPa after HIP for EBM), what supports our conclusion that the surface effect is predominant. Even after machining and polishing, neither SLM nor EBM reached the fatigue limit of a wrought bar (800 MPa). Bagehorn et al. [51] obtained 300 MPa for SLM in the as-built state after HT at 1083 K. By various techniques of roughness reduction (blasting, milling, vibratory grinding, or micromachining), up to 775 MPa fatigue limit was achieved.

Therefore, it is obvious that an inherent surface roughness is the most critical. Surface machining is the best solution how to increase a fatigue limit. A further increase is then possible by the application of HIP, which eliminates the internal defects [16,18,35,40]. However, for some applications requiring lightweight porous structures, the elimination of a surface roughness is not possible, so it is necessary to consider a fatigue performance in an as-built state. Lately, some publications dealing with a chemical treatment of 3D printed surface have appeared, which could be an alternative for porous structures treatment [52,53,54,55].

## 5. Conclusions

This paper has compared titanium alloy Ti6Al4V that was prepared by two powder-bed techniques of additive manufacture—SLM and EBM. EBM was studied in an as-built state, while SLM in a heat treated state to obtain comparable microstructures. Specimens were not further treated to focus on the influence of surface and defects inherent to the building process. The main conclusions are following:

- The metallographic analysis proved a very fine two-phase lamellar microstructure with a low amount of porosity (0.5% in maximum) for both SLM and EBM specimens.
- Internal defects were of a different origin. In SLM, insufficient melting defects prevailed, while spherical pores in EBM resulted from gas entrapment.
- Regarding comparable microstructures and relative densities, static properties in tension reached similar values.
- On the contrary, a significant difference was registered in fatigue behavior. Due to a higher surface roughness and more harmful defects distributed across the whole section, fatigue strength reached 115 ± 13 MPa for EBM when compared to 220 ± 24 MPa for SLM.
- From the fatigue point of view, in the current state of art, SLM seems to be a better choice for the fabrication of porous structures in which surface effect cannot be eliminated.

## Figures and Tables

**Table 1:** Microstructural features compared for SLM and EBM specimens.

Technology	Average Porosity (%)	Pore Density (mm^−2^)	Feret Diameter of Pores (µm)	Prior-β Grains Thickness (µm)	α-Lamellae Thickness (µm)
SLM	0.37	5.0 ± 1.9	34.1 ± 27.9	81 ± 22	0.86 ± 0.27
EBM	0.15	1.6 ± 0.9	42.0 ± 32.6	58 ± 16	0.45 ± 0.13

**Table 2:** Comparison of tensile properties between SLM and EBM.

Specimen	Yield Strength (MPa)	Ultimate Tensile Strength (MPa)	Elongation (%)
SLM	1010 ± 18	1045 ± 12	8.0 ± 0.3
EBM	1074 ± 14	1132 ± 11	7.2 ± 0.2

**Table 3:** Literature review on mechanical properties (YS = yield strength, UTS = ultimate tensile strength) of Ti6Al4V alloy in tension obtained for EBM, SLM followed by heat treatment (HT) and wrought state (MA = mill-annealed, STA = solution-treated + aged).

	Reference	YS (MPa)	UTS (MPa)	Elongation (%)
EBM	our study	1074 ± 14	1132 ± 11	7.2 ± 0.2
	Hrabe (2017) [31]	990 ± 50	1060 ± 20	14 ± 5
	Zhai (2016) [32]	1026 ± 25	1094 ± 21	13 ± 2
	Galarraga (2017) [33]	1001 ± 25	1073 ± 28	11 ± 2
	Murr (2009) [34]	1150	1200	25
	Gong (2015) [17]	962 ± 4	1012 ± 3	8.8 ± 1.6
	Greitemeier (2016) [18]	869 ± 7	965 ± 5	6 ± 0
SLM + HT	our study	1010 ± 18	1045 ± 12	8.0 ± 0.3
	Leuders (2013) [35]	1040 ± 30	962 ± 30	5 ± 2
	Thöne (2012) [36]		~1040	~5.1
	Rekedal (2015) [37]	862 ± 3	937 ± 4	11.4 ± 0.8
	Greitemeier (2016) [18]	1017 ± 7	1096 ± 7	12.0 ± 0.5
	Vilaro (2011) [24]	965 ± 16	1046 ± 6	10 ± 1
	Xu (2015) [25]	1106 ± 6		11.4 ± 0.4
	Vrancken (2012) [29]	955 ± 6	1004 ± 6	13 ± 1
wrought	Murr (2009) [34]	1195 ± 35	1260 ± 42	13 ± 1
wrought + MA	ASM [38]	945	1069	10
wrought + STA	ASM [38]	1103	1151	13
